# The influence of Covid-19 on publications in economics: bibliometric evidence from five working paper series

**DOI:** 10.1007/s11192-022-04473-9

**Published:** 2022-08-12

**Authors:** Constantin Bürgi, Klaus Wohlrabe

**Affiliations:** 1 School of Economics, University College Dublin, Belfield, Dublin 4, Ireland; 2 ifo Institute for Economic Research, Poschingerstr. 5, 81679 Munich, Germany

**Keywords:** Covid, Corona, Pandemic, Citations, A10, A12, I00

## Abstract

We compare Covid-related working papers in economics to non-Covid-related working papers in four dimensions. Based on five well-known working papers series and data from the RePEc website, we find that Covid papers mainly cover topics in macroeconomics and health, they are written by larger teams than non-Covid papers, are more often downloaded and they receive more citations relative to non-Covid papers.

## Introduction

Covid-19 constitutes a dramatic exogenous shock affecting almost all areas of life. It immediately led to an explosion in research, particularly in medicine (Haghani & Bliemer, 2020; Pal, 2021). It also led to an increase in social science research and more specifically economics in order to assess the consequences of the pandemic with an enormous number of new working papers sprawling.

We want to evaluate how these Covid-19-related working papers in economics compare to non-Covid papers published in a number of ways. The analyses by Homolak et al. (2020), Aviv-Reuven and Rosenfeld (2021) and Cai et al. (2021) show across fields that Covid papers are written by fewer authors and with less international collaboration. In economics, both Nagy et al. (2021) and Mahi et al. (2021) provide an overview how the number of Covid-related papers increased. Biondi et al. (2021) detected an increase in journal submissions and fewer female reviewers being available while Kruger et al. (2020) in addition found that women saw a smaller increase in their productivity than men which is also supported by Deryugina et al. (2021), Myers et al. (2020), Amano-Patiño et al. (2020), Squazzoni et al. (2021).

We want to assess whether economics also saw less collaboration and which research topics based on Journal of Economic Literature (JEL) codes were of particular interest since the outbreak of Covid-19. We also investigate two metrics after the working paper has become available, namely the number of citations and downloads of the working papers. Fraser et al. (2021) found that there is a dramatic increase in the number of citations and sharing of Covid-related papers in general science.

In order to assess the impact of Covid-19, we use data from five (major) working paper series in economics obtained by scraping the RePEc website. We provide some descriptive statistics and regression-based evidence to answer how the different metrics differ for Covid-related research relative to non-Covid research. We focus on working papers as they are a rapid communication channel and they are quite common and have long standing tradition in economics.^1^ Furthermore, the publication process in economics is quite slow (Ellison, 2002) and it takes sometimes quite long until a working paper has been published in a refereed journal (Baumann & Wohlrabe, 2020a).

While we focus on some aspects of the publication process from a producer perspective of research, there have also been important changes to the reviewing process and how research is conducted. The wealth of new papers and impacts on the everyday live has affected the reviewing process. For example, Kodvanj et al. (2022) and Aviv-Reuven and Rosenfeld (2021) show that Covid related articles have been processed faster during peer review. Furthermore, there have been a number of retractions and corrections in response to the impacts, see, e.g., Haunschild and Bornmann (2021), Soltani and Patini (2020), Moradi and Abdi (2021), Bagdasarian et al. (2020) or Benjamens et al. (2021). In addition, what research has been published has also changed during the pandemic. In the beginning, it was mainly secondary studies (Di Girolamo and Reynders, 2020) that were not specifically about Covid but adapted to include Covid. Only later, primary studies that are specifically about Covid emerged.

## Data

We collected all working papers made available between 2015 and August 2021 in the following five working paper series:

- NBER (National Bureau of Economic Research) Working Papers
- CEPR (Centre for European Policy Research) Discussion Papers
- IZA (Institut für die Zukunft der Arbeit) Discussion Papers
- CESifo (Center for Economic Studies) Working Papers
- MPRA (Munich Personl RePEc Archive) Working Papers

The first four series are network based, meaning that the authors need to be part of the respective network while anyone can make working papers available in the last one. This choice is driven by the number of yearly published papers, reputation and influence (Baumann & Wohlrabe, 2020). Note that the network membership only affects authors and not potential users, meaning that working papers are freely available independently of network membership. In case of the MPRA working papers, there are no restrictions concerning who is submitting. The original intention was to provide a platform for authors who do not have access to institutional working paper series.

For each paper, we collected the title, the publication year/month, keywords, abstract, JEL codes, authors, downloads and citations from the RePEc website. The RePec website only provides the publication year and not the publication month directly. However, as it provides monthly download statistics, we are able to infer the publication month based on the first month where download figures are reported.^2^ For a few papers, citations and downloads were not available. Therefore we could not define the exact publication date with respect to the month but we have the publication year. Additionally, Baumann and Wohlrabe (2020) have shown that many papers are published in several working paper series simultaneously. In our sample this applies to 2034 papers. We merge the multiple papers into one paper by summing up the number of downloads across versions. RePEc consolidates citations over different versions but often with a time lag. Therefore, we take the maximum of citations across versions as in Wohlrabe and Bürgi (2021). The merged paper is then assigned in a hierarchical way to one of the working paper series using the order listed above. For example, a paper that was in all five working paper series will only show up in NBER once merged. Our results are robust to using a random assignment or reverse ordering.

In order to identify Covid and non-Covid papers, we searched the keywords, abstract and title for the three terms “Corona”, “Covid” and “pandemic”. If at least one of the three terms was found, we deemed the paper to be a Covid paper. Table 1 provides an overview over this sample. The sample refers to unique working papers, i.e., papers appearing in several working papers series are counted only once.

**Table 1:** Working paper sample overview

	All (2015–2021)	Since January 2020	Covid papers	Share Covid papers
Full sample	47,261	9463	1446	0.15
CESifo	5354	1240	171	0.15
NBER	10,639	2530	446	0.14
CEPR	6135	1439	176	0.18
IZA	6608	1404	230	0.12
MPRA	18,525	2850	423	0.16

This table shows the number of working papers included in our sample broken down according to the type of paper identified and the working paper series. The numbers refer to unique working papers, i.e, no double counting

**Fig. 1:**
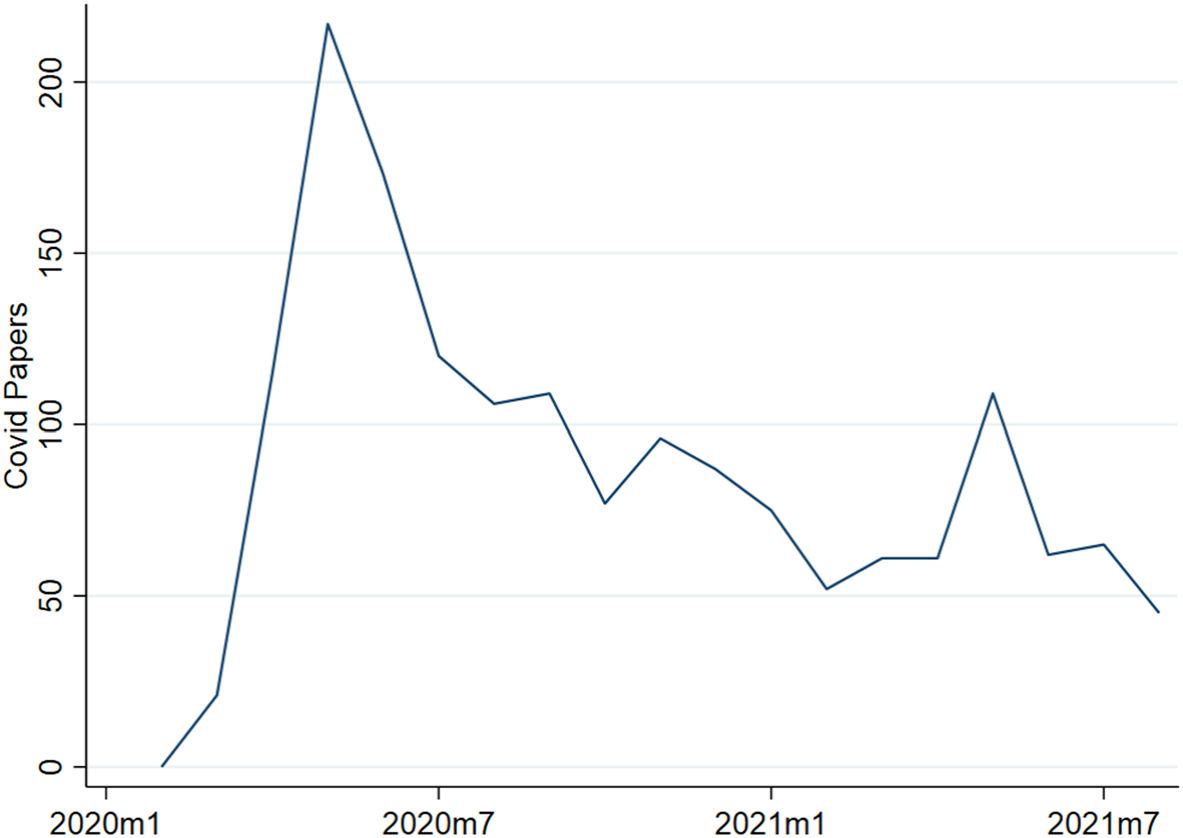
Quantitative development of Covid-related working papers over time.This Figure shows the evolution of the number of Covid-related working papers published

We have around 47 thousand papers in our sample, where about 20% of which were made available since January 2020. Of these papers, 15% were Covid-related papers. This share varies between 12 and 18% across series. Comparing the number of working papers released at a given point in time, we find that most were released in May 2020 with 174 and there has been a steady decline since then as Fig. 1 shows.

## Results

### Topics covered

In a first step, we compare the topics of the different papers based on the Journal of Economic Literature (JEL) codes. In economics, the assignment of publications to sub-fields has a long history. Early classification attempts by the American Economic Association go back to the beginning of the 20th century when ten major categories were defined in the American Economic Review. These categories were developed to arrange publications to their subject matter and have subsequently been revised several times and transferred to the EconLit system, including JEL codes. The majority of economics journals ask authors to provide JEL codes for their papers. Cherrier (2017) provides a detailed overview of the history and meaning of JEL codes. In its current form (since 1991) all JEL codes - the main categories - are designed as ’Exx’, i.e. a letter plus two stages of subcategories indicated by numbers (see https://www.aeaweb.org/jel/guide/jel.php). We focus on the 20 main categories represented by the letters.

For each JEL category, we add up all the JEL codes listed in the papers and divide their sum by the total number of papers. As many papers have multiple codes (2.14 on average), the fractions do not add up to one. For the pre-crisis period we take the average of JEL distribution over the period 2015-2019 in order to avoid to average out short-run developments. Comparing the pre-crisis (column two) period with all papers from the crisis period (column three) there are only minor differences.^3^ There is a notable increase of six percentage points for the health section (JEL code I). Comparing Covid-papers with non-Covid papers within the crisis period, one can spot several clear differences. The two topics with the largest difference for Covid papers (column three) relative to the other papers (column four) are macroeconomics (E) and health/education (I). Whereas the latter on is not surprising, macroeconomics might reflect the impact on the overall economy.

Conversely, it appears that microeconomics and economic development saw a clear dip in their share for Covid papers, even if the overall share (column three) was not much affected. One explanation for this finding could be that researchers in these fields did not switch to writing Covid-related papers to the same extent as other subject fields like macroeconomics and health.

Looking at the development of JEL codes over time from February 2020 to August 2021 there are no obvious trends in the JEL distribution, i.e. the chosen topics, defined by the assigned JEL codes, remained fairly the same across time.

As the share of health paper has increased considerably, we want to take a closer look at these papers. We collected all corresponding co-JEL codes of a paper, i.e. codes that are assigned in addition to the JEL code I for health. We compare the distribution before and after the outbreak of the pandemic. In Table 3 we show the results. Without distinguishing between Covid and non-Covid papers there are no substantial differences of the co-JEL code distribution. By comparing them in the last two columns there are two main issues. First, for there is an increase of macro-related topics (JEL Code E) for Covid papers which goes in line with the results presented in Table 2. Second, labour and demographic issues (JEL Code J) have been more seldomly assigned to health papers in case of non-Covid papers.

**Table 2:** JEL-Code comparison

JEL code	Description	2015–2019	Post January 2020		
			All	Covid-papers	Non-Covid papers
A	General economics and teaching	0.02	0.02	0.02	0.02
B	History of economic thought, methodology, and heterodox approaches	0.02	0.01	0.01	0.01
C	Mathematical and quantitative methods	0.21	0.18	0.15	0.18
D	Microeconomics	0.21	0.24	0.18	0.25
E	Macroeconomics and monetary economics	0.19	0.20	0.27	0.19
F	International economics	0.15	0.13	0.11	0.14
G	Financial economics	0.15	0.16	0.13	0.16
H	Public economics	0.14	0.15	0.19	0.15
I	Health, education, and welfare	0.16	0.22	0.47	0.18
J	Labour and demographic economics	0.22	0.24	0.22	0.25
K	Law and economics	0.04	0.04	0.02	0.04
L	Industrial organization	0.10	0.10	0.07	0.11
M	Business administration and business economics; marketing; accounting; personnel economics	0.05	0.04	0.03	0.05
N	Economic history	0.05	0.05	0.04	0.05
O	Economic development, innovation, technological change, and growth	0.18	0.18	0.14	0.18
P	Economic systems	0.03	0.03	0.02	0.03
Q	Agricultural and natural resource economics; environmental and ecological economics	0.09	0.08	0.04	0.09
R	Urban, rural, regional, real estate, and transportation economics	0.06	0.06	0.06	0.06
Y	Miscellaneous categories	0.00	0.00	0.00	0.00
Z	Other special topics	0.03	0.03	0.04	0.03

This table shows the fraction of working papers with a JEL code corresponding to each category. Note that the fractions add to more than unity as many working papers have multiple JEL codes

**Table 3:** Co-JEL codes for health related working papers

JEL code	Description	2015–2019	Post January 2020		
			All	Covid-papers	Non-Covid papers
A	General economics and teaching	0.02	0.01	0.01	0.01
B	History of economic thought, methodology, and heterodox approaches	0.01	0.00	0.00	0.00
C	Mathematical and quantitative methods	0.13	0.13	0.13	0.15
D	Microeconomics	0.18	0.20	0.21	0.19
E	Macroeconomics and monetary economics	0.03	0.07	0.04	0.15
F	International economics	0.03	0.04	0.02	0.07
G	Financial economics	0.03	0.04	0.04	0.04
H	Public economics	0.17	0.21	0.19	0.24
I	Health, education, and welfare	1.00	1.00	1.00	1.00
J	Labour and demographic economics	0.39	0.35	0.42	0.21
K	Law and economics	0.04	0.04	0.04	0.02
L	Industrial organization	0.04	0.05	0.05	0.05
M	Business administration and business economics; Marketing; accounting; personnel economics	0.02	0.01	0.02	0.01
N	Economic history	0.04	0.04	0.04	0.05
O	Economic development, innovation, technological change, and growth	0.16	0.15	0.17	0.11
P	Economic systems	0.02	0.02	0.01	0.02
Q	Agricultural and natural resource economics; Environmental and ecological economics	0.04	0.04	0.05	0.03
R	Urban, rural, regional, real estate, and transportation economics	0.04	0.04	0.04	0.06
Y	Miscellaneous categories	0.00	0.00	0.00	0.01
Z	Other special topics	0.04	0.04	0.03	0.05

This table shows the fraction of working papers with a JEL code corresponding to each category. Note that the fractions add to more than unity as many working papers have multiple JEL codes

### Number of authors

**Fig. 2:**
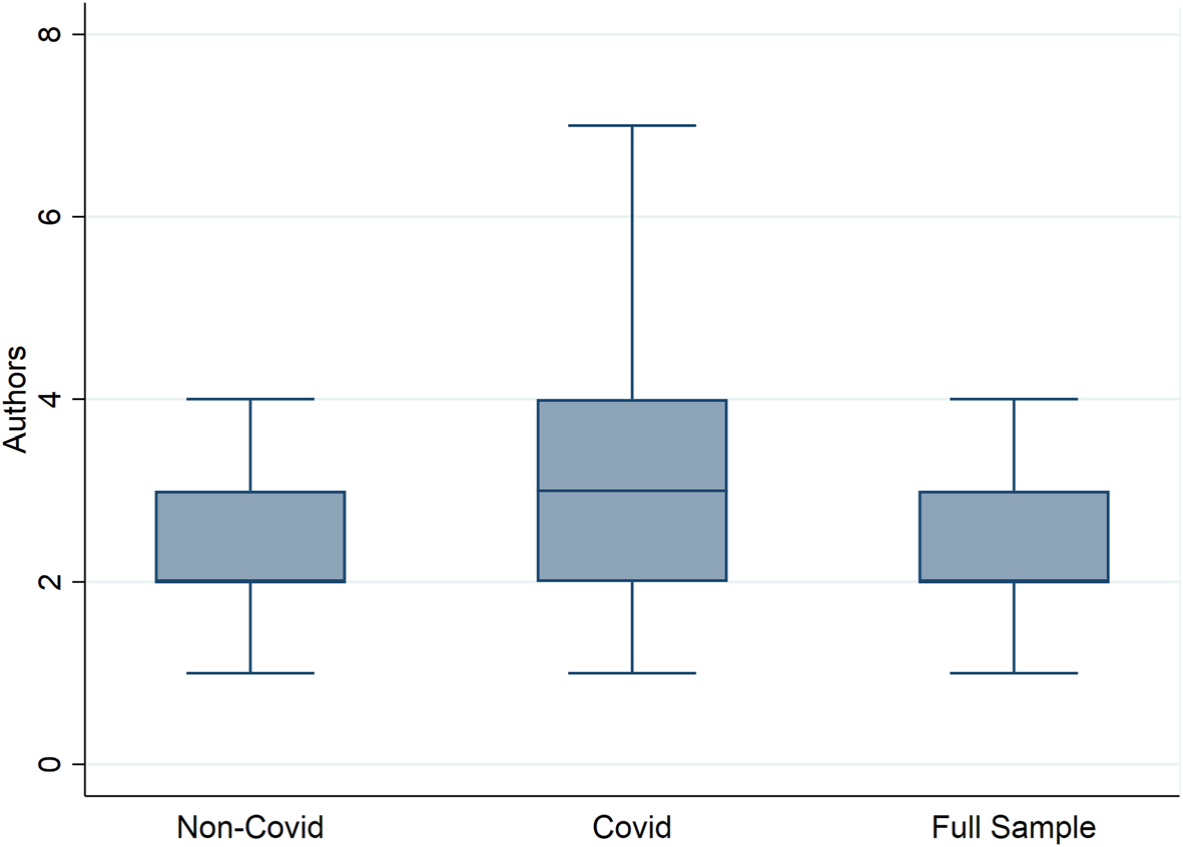
Boxplot comparison for number of authors. This Figure shows the distribution of the number of authors for Covid-related and unrelated working papers published since January 2020. Note that the median is equal to 2 for the full sample and the non-Covid papers of the groupings. For readability we do not plot the outliers

Next, we compare the number of authors that each paper has. We can think of two opposing forces regarding the number of authors. On one hand, the lock-downs and travel restrictions make it harder to collaborate with other authors. This could in turn lower the number of authors for each paper as evidenced in Homolak et al. (2020), Aviv-Reuven and Rosenfeld (2021) and Cai et al. (2021) for general science papers. At the same time, authors might have wanted to publish their research as soon as possible in order to be the first to make a working paper available. The collaboration of several authors can allow the paper to be completed faster and hence Covid papers might have more authors as a result. Table 4 provides some descriptive statistics. Covid-related papers are written on average by 0.3 more authors compared to non-Covid papers. The general relationship holds across all series except the MPRA series, where the Covid papers are written by fewer authors on average. The largest team comprised 43 authors. In Fig. 2, we show the distribution of papers graphically using boxplots. The median paper has 2-3 authors and Covid papers have a much higher variance in the number of authors than other papers. If we take a closer look at papers with a health JEL code (I) we see a similar picture. Covid papers are written on average by 3.1 authors, whereas non-Covid papers by 2.9.

**Table 4:** Number of authors—basic descriptive statistics

	All				Non-Covid				Covid			
	Mean	Std	Min	Max	Mean	Std	Min	Max	Mean	Std	Min	Max
Full Sample	2.6	1.4	1	43	2.6	1.3	1	22	2.9	2.2	1	43
CESifo	2.7	1.1	1	13	2.7	1.1	1	13	3.0	1.3	1	7
NBER	3.0	1.6	1	27	2.9	1.2	1	16	3.5	2.5	1	27
CEPR	2.8	1.1	1	14	2.8	1.0	1	7	3.0	1.5	1	14
IZA	2.9	1.7	1	43	2.8	1.2	1	16	3.3	3.0	1	43
MPRA	2.0	1.3	1	22	2.0	1.3	1	22	1.8	1.1	1	8

This table shows the summary statistics of working papers released since January 2020, broken down by working paper series

In order to check whether these difference are statistically significant after controlling for working paper and topic fixed effects in the form of dummy variables we run three regression models. The first is an ordinary least squares (OLS) regression. As we deal with count data we also consider a Poison regression model and a negative binomial regression (NBREG) model. The variable of interest is a dummy variable which takes a value of one in case of a Covid-related paper and zero otherwise. The top panel of Table 5 shows the regression results. The coefficients of Covid-related papers dummy are positive and highly statistically significant across all three regression models. Thus, our results stand in contrast to the natural sciences, where the number of authors have decreased for Covid-19 papers. If papers with five or more authors (95% percentile) are excluded as shown in the bottom panel, there is no relationship between Covid-papers and the number of authors anymore. This suggests that the results are driven by papers with many authors. Based on the distribution shown in Fig. 2, this result is to be expected as Covid papers are more affected by this restriction than other papers. In both panels in Table 5 the R-squared is rather low which points to the issue that there are more drivers of co-authorship that could not be considered here.

**Table 5:** Numbers of authors—regression results

	OLS	Poisson	NBREG
Panel A: Full sample			
Covid papers	0.24***	0.09***	0.09***
	(0.05)	(0.02)	(0.02)
Observations	9463	9463	9463
R-squared	0.11		
JEL FE	YES	YES	YES
Series FE	YES	YES	YES

Panel B: Excluding articles with 5 and more authors (95% quantile)			
Covid papers	− 0.04*	− 0.00	− 0.00
	(0.02)	(0.01)	(0.01)
Observations	8874	8874	8874
R-squared	0.18		
JEL FE	YES	YES	YES
Series FE	YES	YES	YES

Robust standard errors in parentheses; *** p < 0.01, ** p < 0.05, * p < 0.1

### Downloads

**Fig. 3:**
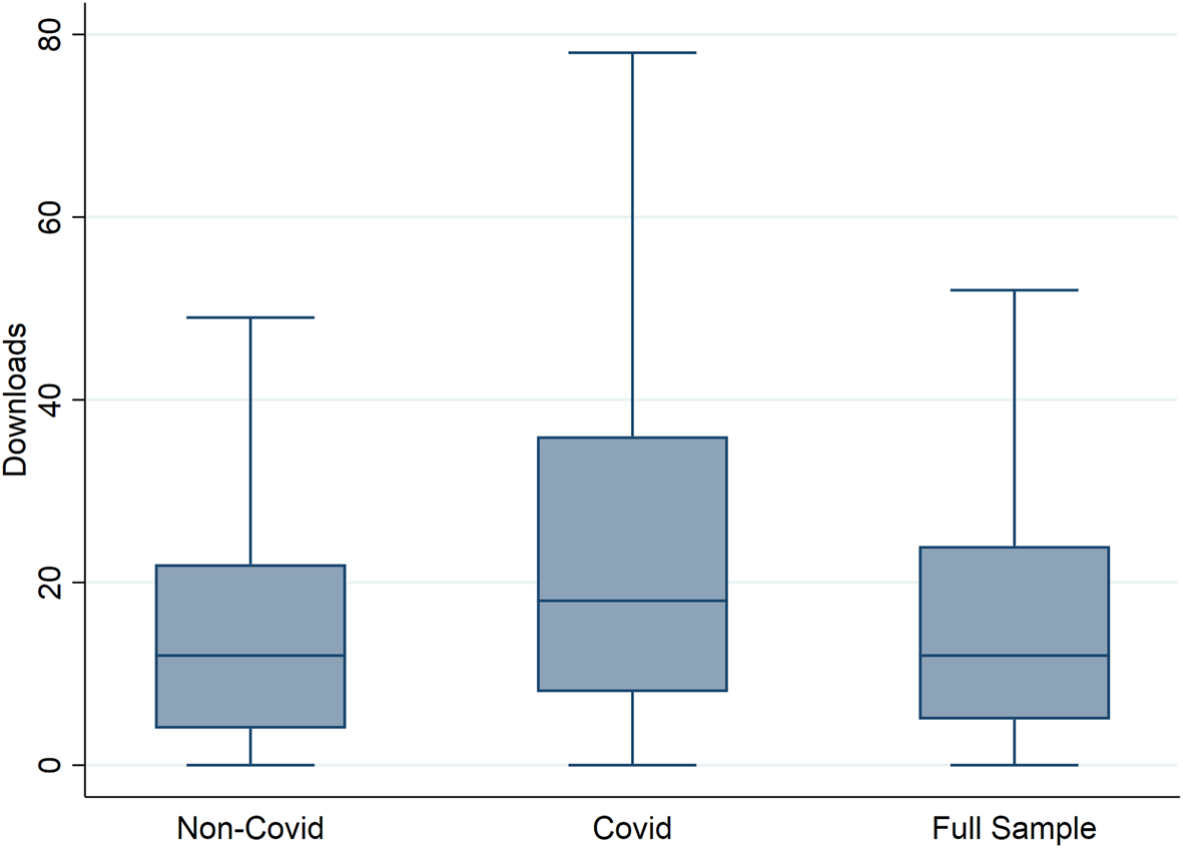
Boxplot comparison for number of downloads. This Figure shows the distribution of the number of downloads for Covid-related and unrelated working papers published since January 2020

Next, we address how many downloads articles receive depending on whether they were Covid-related or not. As shown in Table 6, Covid papers generally get downloaded approximately twice as often compared to non-Covid papers. The most downloaded paper is Sforza and Steininger (2020) with 1116 downloads. CESifo and NBER working papers are downloaded more often on average than the other three series on RePEc. In Fig. 3, we show the corresponding boxplots. Generally, the distribution of downloads is very skewed. Covid papers have a much higher variability in the number of downloads than other papers.

For downloads, the log OLS and negative binomial regressions are of particular importance as the distribution is very skewed. The first two columns of Table 7 show the results for total downloads without taking into account that some articles have been released earlier than others. The Covid dummy is highly statistically significant. In the OLS case a Covid paper receives, ceteris paribus, 17 downloads more than non-Covid papers. In the last column we normalized the downloads by dividing the total downloads by the number of months an article has been made available as a working paper. The results remain robust to this measure as the last column of Table 7 shows.^4^

As the lower panel in Table 6 shows, our results are robust to excluding frequently downloaded papers, again, based on the 95% quantile. While the coefficients become somewhat smaller, they remain highly significant. Based on the distribution shown in Fig. 3, this result is to be expected as Covid papers are more affected by this restriction than other papers.

**Table 6:** Number of downloads—basic descriptive statistics

	All				Non-Covid				Covid			
	Mean	Std	Min	Max	Mean	Std	Min	Max	Mean	Std	Min	Max
Full sample	21.8	36.7	0	1116	19.2	23.2	0	724	36.0	75.0	0	1116
CESifo	29.1	46.2	0	1116	26.6	28.5	0	262	44.6	100.6	0	1116
NBER	28.7	40.6	0	856	25.9	29.8	0	724	41.8	70.9	0	856
CEPR	15.3	21.3	0	369	13.9	17.1	0	190	25.3	38.7	0	369
IZA	16.3	17.7	0	247	14.7	12.7	0	148	24.8	31.7	0	247
MPRA	18.4	39.9	0	966	15.2	18.1	0	413	36.8	92.1	0	966

This table shows the summary statistics for the number of downloads of working papers released since January 2020, broken down by working paper series

**Table 7:** Numbers of downloads—regression results

	Total downloads			Normalized downloads
	OLS	OLS Log Citations	NBREG	OLS
Panel A: Full sample				
Covid papers	16.90***	0.35***	0.68***	0.95***
	(2.10)	(0.03)	(0.06)	(0.16)
Authors	0.23	0.02**	0.01	0.03
	(0.25)	(0.01)	(0.01)	(0.03)
Observations	9343	9343	9343	9343
R-squared	0.07	0.14		0.06
JEL FE	YES	YES	YES	YES
Series FE	YES	YES	YES	YES

Panel B: Excluding papers with more than 62 downloads (95% quantile)				
Covid papers	2.80***	0.16***	0.19***	0.15**
	(0.45)	(0.03)	(0.03)	(0.08)
Authors	0.28***	0.02**	0.02**	0.04
	(0.10)	(0.01)	(0.01)	(0.02)
Observations	8851	8851	8851	8851
R-squared	0.10	0.12		0.06
JEL FE	YES	YES	YES	YES
Series FE	YES	YES	YES	YES

Robust standard errors in parentheses; *** p<0.01, ** p<0.05, * p<0.1

### Citations

**Fig. 4:**
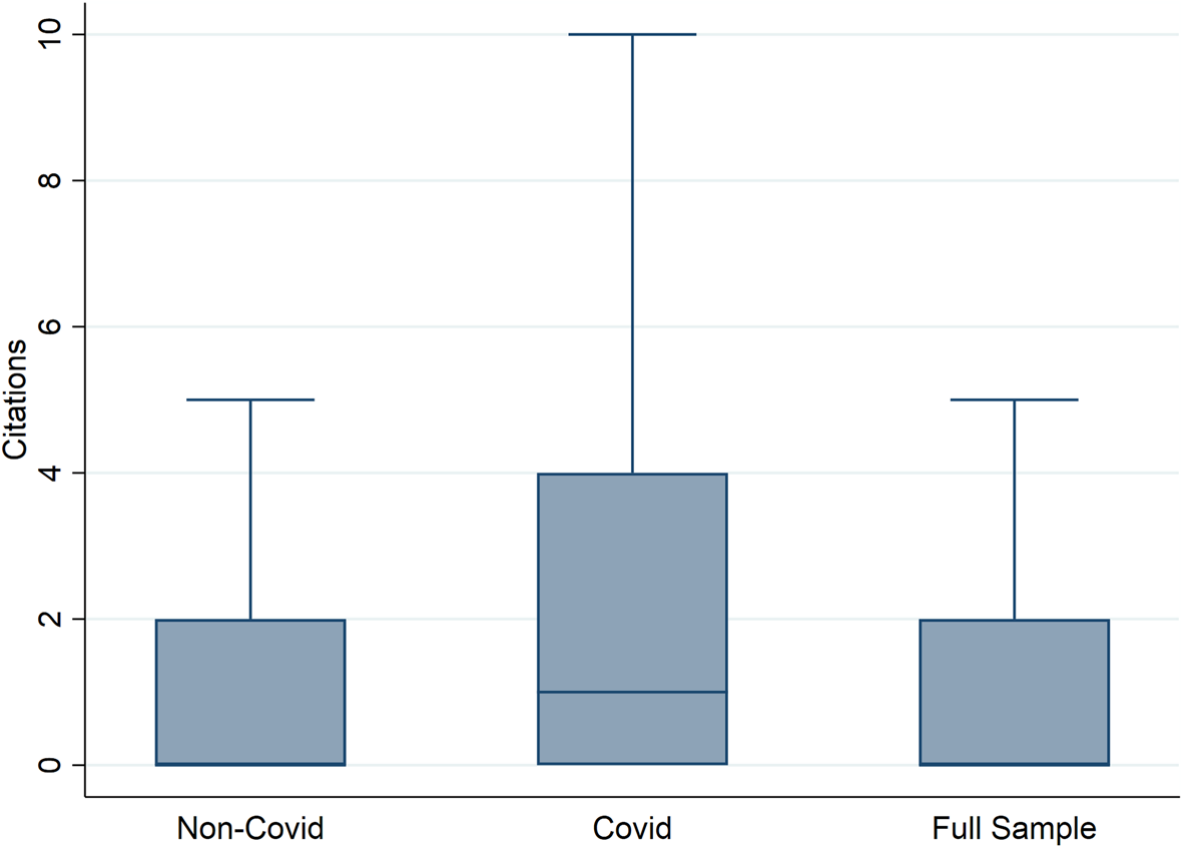
Boxplot comparison for number of citations. Note: This Figure shows the distribution of the number of citations for Covid-related and unrelated working papers published since January 2020. Note that the median is equal to 0 for two of the groupings. For readability we do not plot outliers

Last but not least, we assess whether the citations of the Covid-related papers are higher or lower than non-Covid papers. Table 8 shows that Covid papers received on average six citations more than non-Covid ones. The differences is even more pronounced in case of the NBER working paper series, where Covid-related papers got almost 12 citations more. In Fig. 4, we show the distribution of papers graphically. The median paper has 0-1 citations and Covid papers have a much higher variability in the number of citations than other papers.

Table 9 reports the corresponding regression results. As the distribution of citations is often skewed (Seiler & Wohlrabe, 2014) we also run an OLS regression with logged citations as a dependent variable. Controlling for the number of authors and JEL and working paper series fixed effects, we find a strong statistically significant effect. The number of authors increases the number of citations as typically found in the literature. The $R^2$ is rather low which points to the fact that there are many other potential factors missing (Tahamtan & Bornmann, 2019). As with the downloads, we normalize citations as well to take into account the age of the working paper. Specifically, we divide the number of citations by the number of months a paper has been available. Our results remain robust to this normalization as evidenced by column 4 in Table 9.

Our results are robust to excluding highly cited papers based on the 95% percentile as shown in the lower panel of Table 9. While the coefficients become somewhat smaller, they remain highly significant.

**Table 8:** Number of citations—basic descriptive statistics

	All				Non-Covid				Covid			
	Mean	Std	Min	Max	Mean	Std	Min	Max	Mean	Std	Min	Max
Full Sample	1.7	8.5	0	286	0.8	3.2	0	199	6.7	19.3	0	286
CESifo	1.9	8.4	0	208	1.0	2.5	0	25	7.1	20.2	0	208
NBER	3.5	13.9	0	286	1.4	5.2	0	199	13.2	29.0	0	286
CEPR	1.4	5.2	0	78	0.8	2.3	0	40	5.4	12.5	0	78
IZA	1.3	4.8	0	69	0.6	1.7	0	33	5.1	10.4	0	69
MPRA	0.5	1.8	0	41	0.4	1.5	0	26	0.9	3.1	0	41

This table shows the summary statistics for the number of citations of working papers released since January 2020, broken down by working paper series

**Table 9:** Numbers of citations—regression results

	Total citations			Normalized citations
	OLS	OLS Log citations	NBREG	OLS
Panel A: Full sample				
Covid papers	5.56***	0.62***	1.74***	0.36***
	(0.54)	(0.03)	(0.09)	(0.03)
Authors	0.37***	0.05***	0.20***	0.03***
	(0.09)	(0.01)	(0.02)	(0.01)
Observations	8554	8554	8554	8554
R-squared	0.09	0.09		0.10
JEL FE	Yes	Yes	Yes	Yes
Series FE	Yes	Yes	Yes	Yes

Panel B: Excluding papers with more than 11 citations (95% quantile)				
Covid papers	0.88***	0.29***	0.84***	0.08***
	(0.08)	(0.02)	(0.06)	(0.01)
Authors	0.08***	0.03***	0.13***	0.01***
	(0.02)	(0.01)	(0.02)	(0.00)
Observations	8310	8310	8310	8310
R-squared	0.09	0.09		0.07
JEL FE	Yes	Yes	Yes	Yes
Series FE	Yes	Yes	Yes	Yes

Robust standard errors in parentheses; *** p<0.01, ** p<0.05, * p<0.1

## Conclusion

We have shown that around 15% of economic working papers have been Covid-related between January 2020 and August 2021. This share has decreased since the peak in spring 2020 but there is still a steady flow of Covid papers. When comparing the Covid papers to non-Covid papers made available as working papers at the same time, we find that Covid papers are written by larger teams, are downloaded more often and receive more citations. This somewhat contrasts with general science where articles are written by fewer authors. Further research might be able to determine the exact causes for this divergent pattern.

One important limitation of this paper is that we only looked at the data available on RePEc. While the analysis about JEL codes and the number of authors does not require any additional assumption, the analysis about downloads and citations does. Specifically, there are many places where one can download a working paper including the working paper series website. As a result, we only have a subset of the downloads and need to assume that our sample is representative. Similarly to the downloads, RePEc is somewhat of a closed system regarding citations. Citations are only tracked for papers within RePEc. If for example a medical journal cites an article available on RePEc, this is not counted towards the citation count of the article. As with downloads, we thus need to assume that our data set is representative.

Some potential extensions for future research of our work could include a more granular split into topics using several digits of the JEL code instead of the one digit used here. How the pandemic affected male vs. female researchers differently and last but not least, where the working papers end up. Baumann and Wohlrabe (2020) have found that around two thirds of working papers get published in peer reviewed journals. It would be interesting to see whether this is the case for Covid papers as well and whether the bump in citations from publication is similar to other papers e.g. see (Wohlrabe & Bürgi, 2021).

In the future one could repeat our analysis for articles finally published in a journal. As noted before, the publication process is quite slow in economics. We found only two articles according to our search criteria that were published in the so-called top five journals in economics (Bornmann et al., 2018) that have been published in the last two years.

## Footnotes

1 Working paper or pre-prints series have also been established in the natural sciences like arxiv.org or, more recently, medRxiv.org or bioRxiv.org.

2 Download data in RePEc is available via https://logec.repec.org/.

3 Rath and Wohlrabe (2016) outline the general development of JEL codes over time.

4 As the normalized downloads are not integers anymore, we do not consider the count data models in this case.
